# Thwarting science by protecting the received wisdom on tobacco addiction from the scientific method

**DOI:** 10.1186/1477-7517-7-26

**Published:** 2010-11-04

**Authors:** Joseph R DiFranza

**Affiliations:** 1 Department of Family Medicine and Community Health, University of Massachusetts Medical School, Worcester, MA, USA

## Abstract

In their commentary, Dar and Frenk call into question the validity of all published data that describe the onset of nicotine addiction. They argue that the data that describe the early onset of nicotine addiction is so different from the conventional wisdom that it is irrelevant. In this rebuttal, the author argues that the conventional wisdom cannot withstand an application of the scientific method that requires that theories be tested and discarded when they are contradicted by data. The author examines the origins of the threshold theory that has represented the conventional wisdom concerning the onset of nicotine addiction for 4 decades. The major tenets of the threshold theory are presented as hypotheses followed by an examination of the relevant literature. Every tenet of the threshold theory is contradicted by all available relevant data and yet it remains the conventional wisdom. The author provides an evidence-based account of the natural history of nicotine addiction, including its onset and development as revealed by case histories, focus groups, and surveys involving tens of thousands of smokers. These peer-reviewed and replicated studies are the work of independent researchers from around the world using a variety of measures, and they provide a consistent and coherent clinical picture. The author argues that the scientific method demands that the fanciful conventional wisdom be discarded and replaced with the evidence-based description of nicotine addiction that is backed by data. The author charges that in their attempt to defend the conventional wisdom in the face of overwhelming data to the contrary, Dar and Frenk attempt to destroy the credibility of all who have produced these data. Dar and Frenk accuse other researchers of committing methodological errors and showing bias in the analysis of data when in fact Dar and Frenk commit several errors and reveal their bias by using a few outlying data points to misrepresent an entire body of research, and by grossly and consistently mischaracterizing the claims of those whose research they attack.

## 

In their editorial, Dar and Frenk attempt to defend cherished theories on nicotine addiction from encroaching reality [1]. They challenge the validity of a rapidly growing body of evidence-based clinical data because those data disprove many baseless assumptions that have long been accepted as truths by tobacco researchers. This rebuttal will begin by examining the origins and scientific validity of the theoretical model of tobacco addiction that is reflected in the Diagnostic and Statistical Manual (DSM) [2-4]. This hypothetical model of tobacco addiction will then be contrasted with the real thing as established by replicated, peer-reviewed, clinical studies involving tens of thousands of smokers. A point by point rebuttal to some of the many factual errors, misrepresentations and untenable assertions made in the Dar and Frenk editorial will follow. The objective of this essay is to help readers distinguish between fact and fiction in the literature on tobacco addiction.

## The origins of the received wisdom

The pioneers of tobacco research in the 1960's and 70's could not know how tobacco addiction developed because the first study describing the development of tobacco addiction was published in the year 2000 [5]. However, they did recognize that heavy daily smokers were addicted to tobacco. Starting in the early 1970's a series of articles in prominent medical journals equated tobacco addiction with heavy daily smoking [6-9]. By today's standards, these articles are notable for their many pages of detailed assertions regarding the nature of tobacco addiction which are unsupported by a single reference. These speculations formed the foundation for what would become the accepted wisdom among tobacco researchers for the next 4 decades: the threshold model.

In brief, the threshold model maintains that until tobacco consumption is maintained above a threshold of 5-10 cigarettes per day (cpd) for a prolonged period, smokers are free of all symptoms of tobacco addiction. It holds that declining blood nicotine levels trigger withdrawal symptoms so quickly that addicted smokers must protect their nicotine levels by smoking at least 5 cpd. The threshold model states that until addiction is established with moderate daily smoking, smoking is motivated and maintained by peer pressure, pleasure seeking and the social rewards of smoking. Under the threshold model, escalating consumption over many years is driven by increasing tolerance to the pleasurable effects of nicotine.

The DSM does not reference the threshold model, but restates many of its speculations as fact. For example, for the past 30 years the DSM has represented that moderate daily smoking is a prerequisite for addiction [2-4]. DSM-III asserts that tobacco withdrawal symptoms can be diagnosed only in individuals who use "tobacco for at least several weeks at a level equivalent to more than ten cigarettes per day."[2] DSM-IV states that "daily use of nicotine for at least several weeks" is required for nicotine withdrawal. The DSM provides no references to support any of these statements.

The DSM's assertion that moderate daily smoking is a prerequisite for nicotine dependence was reinforced by a series of studies on "chippers," atypical individuals who smoke fewer than 5 cpd over many years and who were reported to have no symptoms of addiction [10-13]. The assertion that adult chippers had no symptoms of addiction was generalized to indicate that all light smokers are free of addiction. This idea is reflected in a proposal that cigarettes could be rendered non-addictive by reducing nicotine levels in cigarettes to such a degree that smokers would not be able to obtain as much nicotine in one day as they would obtain from smoking 5 normal cigarettes [14]. Given the short half life of nicotine,[15] the idea that smokers must maintain a threshold blood level of nicotine to avoid withdrawal symptoms implies that withdrawal symptoms have a very rapid onset. (If withdrawal symptoms were delayed by a day or two, smokers would not feel compelled to smoke every day.) The presumption of a rapid onset for withdrawal is reflected in DSM-III and DSM-III-R which indicate that withdrawal "symptoms begin within 24 hours of cessation or reduction in nicotine use," but again, no reference is provided [2,3].

The scientific method demands that theories such as the threshold model be tested and rejected if they are not supported by the data. The main tenets of the threshold model can be stated as testable hypotheses.

### Hypothesis 1. Tobacco addiction cannot occur in nondaily smokers, or even in daily smokers who regularly consume fewer than 5 cpd [2]

Although it is difficult to prove a negative, this hypothesis would be supported if study after study demonstrated that all surveyed subthreshold smokers (individuals who smoke < 5 cpd) have no symptoms of addiction. In fact, evidence of tobacco addiction among subthreshold smokers has been reported in every study that has examined the issue [12,16-32]. Even in the largest chipper study, chippers' ratings of their addiction to tobacco averaged 2.7 on a scale from 1 to 5, 48% reported it would be difficult to go without smoking for a week, 65% experienced craving during withdrawal, and smaller proportions experienced the withdrawal symptoms of irritability, nervousness, tension, restlessness and disrupted concentration [12]. Since no studies have demonstrated a complete lack of addiction symptoms in any representative population of subthreshold smokers, the peer reviewed literature soundly refutes the hypothesis that tobacco addiction requires as a prerequisite the daily consumption of 5-10 cigarettes. The threshold model and the DSM are wrong.

### Hypothesis 2. Tobacco addiction requires prolonged daily use as a prerequisite [4]

This hypothesis has been tested by following novice adolescent smokers prospectively to determine if they remain free of addiction symptoms until they have been smoking daily for a prolonged time. The first prospective study of the onset of tobacco addiction reported that two-thirds of the individuals that developed symptoms of addiction did so without smoking daily [5,24]. Many subjects developed symptoms quite soon after the onset of intermittent tobacco use. These findings have been replicated in several longitudinal studies, [24-26,28,29] in cross-sectional studies showing symptoms of addiction in nondaily smokers,[23,27,33,34] and by case histories showing the same [35]. As there are no studies documenting the absence of tobacco addiction in all nondaily smokers, the peer reviewed literature strongly refutes the hypothesis that daily smoking is a prerequisite for tobacco addiction. The threshold model and the DSM are wrong.

### Hypothesis 3. Nicotine withdrawal symptoms begin within 24 hours in all smokers [2,3]

The standard subject in all early smoking studies was an adult who had been a heavy daily smoker for decades. Such individuals do experience nicotine withdrawal soon after their last cigarette [36]. A problem arises when this observation is inappropriately generalized by applying it to all smokers, including children, novices and nondaily smokers. This hypothesis would be supported by a study that demonstrates that all smokers in a broadly representative population either experience withdrawal within 24 hours or not at all. I am aware of only 4 surveys in which unselected populations of smokers were asked to report how long it takes for withdrawal symptoms to appear. The data from all 4 surveys and a case series indicate that withdrawal symptoms take much longer than 24 hours to appear in nondaily smokers [35,37-39]. As no study has demonstrated an absence of delayed withdrawal symptoms in nondaily smokers, the available peer reviewed literature consistently refutes the hypothesis that nicotine withdrawal always begins within 24 hours in all smokers. The threshold model and the DSM are wrong.

### **Hypothesis 4. Addicted smokers must maintain nicotine above a threshold blood concentration to avoid withdrawal**

Heavy smokers smoke in a way that suggests that they are trying to maintain nicotine above a minimal threshold blood concentration [40] (but addicted nondaily smokers do not). Although a threshold blood nicotine concentration appears to be the central premise of the theoretical model that has dominated the field for 40 years, no study has directly tested this hypothesis by measuring withdrawal and nicotine levels while smokers smoke ad lib to determine if they in fact defend a threshold level of nicotine in the blood in response to withdrawal symptoms. Two small studies that measured craving and nicotine levels simultaneously in heavy daily smokers did not report evidence of a threshold level [41,42].

Since a person must smoke at least 5 cpd to maintain a minimum nicotine level throughout the day,[14] another approach to testing this hypothesis would be to determine if all smokers that experience withdrawal symptoms smoke at least 5 cpd. This test has been completed over a dozen times, and always with the same result. Withdrawal symptoms have been reported in smokers of fewer than 5 cpd in every study that has examined this issue [16-30]. As no study has demonstrated a threshold, and every relevant study in the literature indicates that many smokers who experience withdrawal make no attempt to smoke 5 cpd, the central premise of the threshold model is wrong.

### **Hypothesis 5. Psychosocial factors maintain smoking over the several years it may take to reach threshold levels of smoking**

There must be thousands of studies that demonstrate that social factors such as socioeconomic status, smoking by family and friends, cigarette advertising, the availability of cigarettes, smoking depictions in movies, and attitudes and beliefs are predictive of which youth will try smoking [43-46]. However, if such factors sustain tobacco use until tobacco addiction develops, they should predict which smokers will advance to addiction in prospective studies. But this has not been shown. None of more than 40 psychosocial risk factors for the onset of smoking was able to predict the progression to tobacco addiction [47]. The author is aware of no studies that establish that peer pressure of other social factors sustain adolescent or young adult smoking over the 4 or 5 years it may take for smokers to reach threshold levels of smoking.

### **Hypothesis 6. Increasing tolerance to the pleasurable effects of smoking drives the escalation in tobacco use up to the threshold of addiction**

The author is not aware of any studies that demonstrate that smokers must smoke more cigarettes over time to obtain the same amount of pleasure (for example smoking 10 cpd to obtain the same pleasure initially obtained from smoking 1 cpd. Indeed, our data indicate that the pleasure obtained from smoking each cigarette actually increases in proportion to the degree of addiction, with pleasure ratings correlating strongly with addiction severity [39]. While this is only one study, it directly contradicts the hypothesis that non-addicted novice smokers obtain much more pleasure from each cigarette than do addicted heavy smokers.

To summarize to this point, all of the hypotheses that are central to the threshold theory have either been soundly refuted or are directly contradicted by a preponderance of peer-reviewed research. The scientific method demands that these hypotheses be rejected, and yet the threshold theory remains the predominant theory accepted by tobacco researchers. This raises questions regarding why the field would accept the threshold model as an unassailable truth for 4 decades without ever testing its central premises?

### Validity issues with the DSM

As demonstrated by the quotations above, the DSM represents as fact a number of the hypotheses that together make up the threshold model. The DSM has asserted that daily smoking is a prerequisite for addiction, that consumption at a level of 10 cpd is required for withdrawal, and that withdrawal must start within 24 hours. It is not surprising that none of these assertions are supported by references in the DSM, as there does not appear to be a single published study that supports any of these hypotheses. In fact, each of these statements is contradicted by every relevant published study. The appearance of factual errors in the DSM reveals that it is not an entirely evidence-based document. The DSM criteria themselves represent a theory, it is the theory that the 7 criteria accurately describe tobacco addiction and that the presence of 3 of the 7 criteria provides a sensitive and specific diagnostic test for tobacco addiction. What large body of peer reviewed data established the validity of the DSM criteria?

Recently the author had the honor of leading an international team of 14 doctorate-level researchers from a variety of disciplines in an evaluation of the validity of the DSM and International Classification of Diseases (ICD) tobacco addiction diagnostic criteria [48,49]. Applying contemporary standards of scientific rigor, this team critically examined every relevant English language publication from the past 30 years. A defining feature of both the DSM and ICD criteria is that they set a severity threshold for the diagnosis of addiction. Under either paradigm, smokers must have at least 3 diagnostic criteria to earn a diagnosis. Our search failed to locate a single study that established the validity of either the DSM or ICD threshold [48,49]. Only 3 studies could address the validity of a diagnostic threshold and none found any evidence that a threshold exists [50-52]. The historical record indicated that the decision to use a 3-symptom threshold was not evidence-based: "the [DSM-IV] work group increased the requirement for dependence to a minimum of four from three criteria and decreased it to two; these changes greatly increased and reduced the proportion of users with reported problems who met criteria for dependenceâ€¦ After consideration, three continued to be judged as the most appropriate for meeting dependence."[53] This record indicates that the 3-criteria threshold was not set by comparing the diagnostic criteria to a real measure of tobacco addiction in a clinical population. Rather, the committee decided what proportion of smokers they would want to label as addicted, and like Goldilocks, they tried different thresholds until they found the one that was just right.

The historical record indicates that at the time of their initial publication, neither the DSM nor the ICD criteria were based on any identifiable body of smoking research other than a few studies relating to the single criterion for withdrawal. After a thorough consideration of both sets of criteria in relation to contemporary psychometric standards, the consensus was that neither set of diagnostic criteria had been validated prior to its publication, nor at anytime during the intervening 30 years [48,49]. The DSM and ICD diagnostic criteria rested solely on the credibility of the issuing organization as there is not a single study that demonstrates that either set of criteria represents a sensitive and specific diagnostic tool. The DSM and ICD criteria represent unproven hypotheses that these criteria accurately diagnose tobacco addiction.

### Who gets to define addiction?

What few people realize is that the DSM and ICD represent suggested nomenclatures, a set of definitions intended to foster clearer communication among researchers and practitioners. The criteria encourage a common usage of language but the definitions do not reflect the outcome of scientific studies that establish the true nature of tobacco addiction. They are not a distillation of all human knowledge regarding tobacco addiction; they represent a gentlemen's agreement on vocabulary.

Some researchers accept that tobacco addiction is whatever the American Psychiatric Association or the World Health Organization say it is, but that is not how the scientific method works. The DSM and ICD cannot "define" the characteristics of tobacco addiction; nature defines the characteristics of tobacco addiction. At best, mankind can accurately describe what nature produces. The burden of proof for the DSM and ICD is to show that they accurately reflect the characteristics of tobacco addiction as revealed by clinical studies of real smokers. This they have failed to do [48,49]. Dependence as defined by DSM has little resemblance to what smokers identify as addiction. DSM-III dependence shows a poor correlation (r = .30) with self-assessed addiction and DSM-IV does not do much better (r = .48) [54]. On general principles Dar and Frenk dismiss outright the idea that smokers can assess their own symptoms, and yet self-rated addiction shows an excellent correlation with self-rated difficulty quitting (r = .89), and correlates better with all other indicators of dependence than does the DSM [54]. The hypothetical construct that is measured by DSM appears to have little in common with what smokers experience as addiction. The DSM fails this test of construct validity.

Neither the DSM nor the ICD describes or explains the clinical course of tobacco addiction, i.e., the manner in which symptoms evolve over time. The most clinically relevant feature of tobacco addiction is that smokers fail in their attempts to quit smoking. DSM dependence has no apparent relevance to this aspect of tobacco addiction. About 90% of smokers relapse when they make an unassisted attempt to quit,[55] yet the DSM diagnoses only about half of smokers to be dependent [56]. In one study, one-third of current smokers who had failed to quit in 6 or more attempts were not dependent according to DSM-IV [57]. It has not been shown that the DSM and ICD diagnostic criteria provide a valid and accurate description of tobacco addiction as experienced by smokers [48,49].

## An evidence-based clinical description of the natural history of tobacco addiction

When I designed the first prospective study to investigate the early development of tobacco addiction, I was faced with a dilemma over how to measure it. I didn't want to repeat the mistakes of DSM and ICD by presuming that I could define what is and is not addiction. To smokers, the defining characteristic of addiction is that they find it difficult to stop. So I created an instrument that asked smokers about a variety of symptoms that would make quitting more difficult or unpleasant, such as craving, feeling addicted and experiencing withdrawal symptoms. Failed quit attempts are an obvious indication that a smoker is experiencing difficulty with quitting. Since anonymous peer reviewers would not allow me to use the words "tobacco dependence" in a manner that contradicted the DSM,[58] I coined a new term, the loss of autonomy, to describe these symptoms. The instrument that was used in this study was later refined through psychometric evaluations to become the 10-item Hooked on Nicotine Checklist (HONC) [59]. The HONC is the most thoroughly validated measure of tobacco addiction, and is in use on 5 continents in 18 languages [30,59-66]. The development of a sensitive, validated measure of salient individual symptoms enabled researchers to study the clinical course of tobacco addiction without making a predetermination of what constitutes a diagnosis of tobacco addiction.

As presaged by the forgoing discussion, the clinical course of tobacco addiction is the opposite of what is described by the threshold theory in virtually every aspect. Every prospective study of the onset of tobacco addiction indicates that symptoms of addiction begin to appear soon after the first cigarette in the most susceptible smokers [5,24-26,28,29]. Symptoms of addiction develop quickly during intermittent smoking, increasing from a prevalence of 25% among those who have smoked only 1 or 2 cigarettes to about 95% among those who have smoked 100 or more cigarettes [33]. The findings from the longitudinal studies are backed by data from large national cross-sectional studies [23,27,33,34]. One such study involved 3 consecutive, representative national surveys in New Zealand involving some 30,000 adolescent smokers [33]. In each consecutive survey, approximately 25% of youth who had just smoked their first cigarette reported symptoms of tobacco addiction, most commonly craving. Symptoms of tobacco addiction, including failed quit attempts, have been reported by nondaily smokers and daily smokers of fewer than 5 cpd in every relevant study [16-32].

Dar and Frenk assert that our conclusion that addiction begins during intermittent smoking depends on an untenable and idiosyncratic definition of tobacco addiction. Whether or not you call it a loss of autonomy, failed quit attempts and nicotine withdrawal symptoms are signs of addiction. There is nothing idiosyncratic about that. The onset of addiction prior to the onset of daily smoking has also been documented using the DSM and ICD as outcome measures (but this requires one to ignore the DSM's erroneous statements regarding daily smoking being a prerequisite for addiction and withdrawal) [25,26]. So, by any measure, tobacco addiction is present in nondaily smokers.

In order to dissuade youth from experimenting with smoking, I have emphasized that symptoms of addiction can appear as early as following the first cigarette in the most susceptible individuals [67,68]. One might reasonably question, as Dar and Frenk do, whether these very early symptoms are clinically important. The clinical importance of these early symptoms is well established. In one 3-year prospective study, youth who reported at least one symptom of lost autonomy were 44-fold more likely to be current smokers at the end of follow-up [24]. In another, youth who reported at least one symptom were 196-fold more likely to progress to daily smoking [25]. Thus, symptom reports after smoking a single cigarette are powerful predictors of the clinical course of the disease. If, as Dar and Frenk allege, symptom self-reports were unreliable, they would not predict future events with odds ratios of 196.

Contrary to the hypothesis that psychosocial factors are the primary motivator of smoking for the first several years, the predictive power of these early symptom reports exceeds that of any psychosocial risk factors by orders of magnitude [47]. The mean smoking frequency at the first appearance of symptoms of lost autonomy is 2 cigarettes per week,[24,25] and smoking at this level at the age of 12 increases the chances of progressing to heavy smoking as an adult 12 years later with an odds ratio of 174 [69]. Any arguments that Dar and Frenk make about theoretical reasons why people cannot be trusted to evaluate their own symptoms are meaningless in the face of empirical data such as these.

There is a physiological explanation for why symptoms that appear after smoking a few cigarettes are excellent predictors of the course of the illness. In every relevant study, subthreshold smokers have reported nicotine withdrawal symptoms,[16-32] and in every relevant study, subthreshold smokers report that the onset of nicotine withdrawal can be long delayed after the last cigarette [35,37-39]. (Anyone who doubts that withdrawal can be delayed by several days, need talk to no more than 2 or 3 "social smokers" to find one who has withdrawal symptoms if they go too many days without smoking.) It is not uncommon for novice smokers to report that they do not experience craving for a cigarette until a few weeks after their last smoke [35,37-39]. Smokers' reports of their latency to the onset of withdrawal are valid and reliable [39].

The latency to the onset of withdrawal places an outside limit on how far apart smokers can comfortably space their cigarettes. Over time, as tolerance develops, the duration of relief from withdrawal that is afforded by smoking a cigarette shortens [39]. As the latency shortens, smokers feel compelled to smoke at more frequent intervals. The progressively shortening latency to withdrawal explains the smooth trajectory in escalating smoking frequency that has been observed in every longitudinal study [70,71]. The reason why early symptoms are capable of predicting future smoking behavior with odds ratios close to 200 [25] is that the addiction is already established when the first symptoms appear. The progressive shortening of the latency to withdrawal makes the escalation of smoking inevitable unless cessation is accomplished.

The shrinking latency to withdrawal explains the escalation from intermittent smoking to daily smoking. It explains the smooth escalation from smoking one cigarette per day to smoking two packs per day. As the latency to withdrawal shrinks to less than the duration of sleep, it explains why addicted smokers feel compelled to smoke as soon as they get out of bed in the morning. The latency to withdrawal shrinks to different degrees and at different rates in different smokers and this explains why some addicted smokers experience withdrawal within 20 minutes of their last cigarette while others can go several hours despite the fact that nicotine metabolism differs little between smokers. A contemporary study of chippers found that every chipper had symptoms of addiction [30]. In chippers, the latency to withdrawal only shortens to a certain extent, allowing them to maintain a relatively low frequency of smoking despite the fact that they experience the same symptoms of addiction as typical smokers [30]. The latency to withdrawal explains why smokers smoke an extra cigarette before entering a venue where they will not be allowed to smoke. Smoking a preemptory cigarette resets the timer on their latency to withdrawal. All of this we know, not from hypothetical models of addiction, but by histories provided by real smokers [35].

Empirical data establish that the threshold theory does not explain a single thing about the behavior of smokers. Empirical data establish that the early development of withdrawal symptoms followed by a shortening of the latency to withdrawal explains a great deal about smokers' daily behaviors from the first cigarette to heavy adult smoking. Dar and Frenk argue that this entire description of the characteristics of tobacco addiction based on studies of tens of thousands of smokers should be ignored by tobacco researchers because it contradicts the DSM.

## Rebuttal

The editorial by Dar and Frenk represents another installment in a series of papers published by Dar in which he attacks the work of other researchers [72-76]. This time Dar and Frenk attempt to discredit the entire body of peer reviewed research that proves that addiction begins quickly. Their debate strategy is to rhetorically link all the relevant research together so that an attack on the weakest link will serve to discredit every other study through guilt by association. They do this by repeatedly referring to a "hooked on nicotine research program" which does not exist, and then accusing selected researchers of bias and methodological errors. The research that establishes that symptoms of addiction appear quickly comes from many independent research groups. There is no coordinated "hooked on nicotine research program." Identifying an imaginary flaw in one study does not prove that every other peer reviewed study in the literature is likewise flawed.

Dar and Frenk argue that the loss of autonomy is so different from the DSM that it is irrelevant to tobacco researchers. By using measures of lost autonomy with real smokers, researchers have been able to develop the first evidence-based description of the characteristics of tobacco addiction as outlined above. It is through the lens of the loss of autonomy that the clinical course of tobacco addiction has been revealed. It is the DSM and ICD that have no demonstrated relevance to the important clinical features of tobacco addiction [48,49].

Dar and Frenk argue that the diagnosis of tobacco addiction should be delayed until 3 DSM criteria are present so that a diagnosis will be more meaningful. They also argue that, in their opinion, researchers were too liberal regarding what is required to meet the ICD standards. Good medical practice strives to diagnose diseases as early as possible to be able to arrest the disease progression. With the HONC, we can now identify tobacco addiction very early in its course. When one can use the HONC to identify novice smokers who are 200 times more likely to advance to daily smoking and perhaps 174 times more likely to advance to heavy smoking in adulthood, what is the clinical advantage of delaying a diagnosis by relying on the DSM? This makes as much sense as delaying the treatment of cancer.

As a physician, I expect every medication I prescribe to start to work with the first dose. I don't understand why Dar and Frenk find it impossible to imagine that nicotine might also start to work with the first dose. I know of no plausible physiological mechanism that would explain why the addictive effect of nicotine would start only after many thousands of doses. What other drug works in this way?

Dar and Frenk argue that difficulty in quitting cannot be used to diagnose tobacco addiction because people also find it difficult to change non-addictive behaviors. They make the common mistake of arguing that a nonspecific symptom cannot be used to diagnose a disease. The DSM made the same mistake when it eliminated craving as a nicotine withdrawal symptom [4]. The fact that a disease symptom can occur in other settings does not mean it cannot be used to make a diagnosis. Fever is used to diagnose malaria even though an elevated temperature can also be caused by exertion in hot weather. Uterine contractions are used to diagnose labor even though they also occur during normal menstruation. Since very few diseases cause pathognomonic symptoms that appear in no other setting, physicians such as myself must diagnose diseases by evaluating symptoms that are not specific to any one disease.

Another rhetorical device used by Dar and Frenk is misrepresentation and exaggeration. They represent to readers that the idea that addiction starts among never smokers or after one puff is the "principal claim of this research program" when in fact these statements have never appeared in any publication. (If anything, the fact that withdrawal is present in nondaily smokers and that the latency to withdrawal onset shortens over time is the most important discovery.) Dar and Frenk focus on a single study in which 10-year-old children were asked to complete a written survey which included the terms 'mental addiction' and 'physical addiction' [77]. Fewer than 2 percent of 1488 ten-year-olds who claimed to have never smoked reported mental or physical addiction to tobacco. The authors of this study pointed out many methodological limitations and were very careful not to assert that youth who had never smoked were addicted to tobacco. Yet according to Dar and Frenk this is the principal claim of not only this study, but all researchers in the field: "as shown above, the "hooked on nicotine" program holds that adolescents can lose autonomy over smoking after smoking a single puff in their lifetime and even when they have only been exposed to secondhand smoke. This leads to the paradoxical conclusion that one can lose autonomy over a behavior (in this case, smoking) that has never been performed."[1] The study in question (1) did not use the Hooked on Nicotine Checklist, (2) never mentioned a loss of autonomy, (3) did not claim that 10-year-olds are adolescents, and (4) never claimed that children who never smoked were addicted to smoking. This is a mischaracterization on the part of Dar and Frenk who exploit these misrepresentations to call into question the validity of all research conducted under the umbrella of the nonexistent "hooked on nicotine program."

First off, the validity and reliability of other measures such as the HONC (which does not include the terms 'mental addiction' and 'physical addiction') is well established [30,59-66]. Obviously, any problems with the terms mental addiction and physical addiction has no relevance to published studies that do not include these items. Second, even if these particular terms were to be found to be unreliable, this has no bearing on the literature that establishes early addiction; only one study included these terms, and only as a tertiary indicator after the HONC and the ICD. Third, these data do not indicate that nonsmokers experience the same symptoms as early smokers. More likely explanations are that 2% of ten-year-olds either cannot read or understand the terms mental addiction and physical addiction, or the children lied about being nonsmokers. So, based on the outlying responses of a few ten-year-olds, Dar and Frenk argue that the data from every other study on early addiction cannot be trusted, including dozens of other studies conducted by independent researchers using unrelated, validated measures.

Focusing on outlying responses from 40 subjects in a survey of over 96,000 students, Dar and Frenk assert that youth who smoked one cigarette "and never smoked again" say that they can't quit. They argue that this is illogical and calls into question the validity of the data from the other 25,000 smokers in the study [33]. Since the study in question, the New Zealand annual Year-10 national smoking survey, is cross-sectional, it is unclear how Dar and Frenk determined that youth who had only smoked one cigarette prior to the survey "never smoked again." In a survey involving 96,000 adolescents, some subjects will complete the survey in the days immediately following their first cigarette. An interpretation of these data that is consistent with other studies, and that does not defy logic, is that these youth had smoked one cigarette and already felt that they needed another. The data from the longitudinal studies indicate that even one such symptom reported early on increases the likelihood of progressing to daily smoking with an odds ratio of 196 [25]. Given these data, subjects' assessments that quitting would be difficult are probably very accurate.

Dar and Frenk accuse Canadian researchers of bias in their scoring of Pierce's validated measure of susceptibility [77]. Concerning responses to 3 items such as "at any time during the next year do you think you will smoke a cigarette?" the researchers included the response "probably not" with the responses "probably yes" and "definitely yes." Dar and Frenk assert that this is evidence of bias, and on that basis call into question the integrity of the entire fictional "hooked on nicotine program." These critics would have been well served to check their facts first. The scoring method that Dar and Frenk cite as evidence of intentional bias is actually the official scoring method that has been used for this popular validated measure since it was published in 1996 [78]. Anyone who had worked in the field of adolescent smoking research would know that.

Dar and Frenk also accuse the same Canadian researchers of a methodological error in identifying youths who have never smoked as being susceptible to initiating smoking. They argue that only youth who have tried smoking are susceptible. Any student of epidemiology knows that only individuals who do not have the disease are considered susceptible, once a person has a disease, they become a case and are removed from the pool of susceptible individuals. The Canadians' usage of the term susceptibility is consistent with its usage in epidemiology and in tobacco research [78]. These factual errors suggest that Dar and Frenk lack the expertise in smoking research to critically comment on this body of work.

An unfathomable assertion by Dar and Frenk is that people who smoke less than once per month cannot be said to have stopped smoking because "these responders were in a virtually permanent state of stopping."[1] This puzzling statement appears to reflect the erroneous assumption that intermittent smoking is without an effect on adolescents and therefore smoking less than once per month is functionally equivalent to not smoking at all. Smoking less than once a month is not permanently stopping, it is the typical pattern of smoking initiation and intermittent smoking predicts an escalation to addiction [79,80].

Dar and Frenk point out that our diagnosis of ICD dependence as early as 13 days after the onset of smoking is inconsistent with the ICD stipulation that symptoms be present for a month. While technically true, the time duration and clustering stipulations in ICD and DSM have never been validated, and have been uniformly ignored by researchers for decades because they are too cumbersome to implement in a survey [48,49]. Are Dar and Frenk arguing that our conclusion that dependence begins quickly is wrong because we should have marked the onset of ICD dependence at 30 days for this subject?

Dar and Frenk fault one study purely on the basis that the results contradict their own preconceived notions: "how could other symptoms required to make the diagnosis (e.g., withdrawal, tolerance, preoccupation with the substance, continued use despite harmful effects) develop in such a brief period?"[1] The fact that the data contradict their preconceived notions does not represent a methodological flaw in the study.

Dar and Frenk argue that "the suggestion that nicotine dependence can be reduced to craving is contradicted by converging lines of empirical evidence. First, craving is not specific to drugs. As smoking combines (and therefore confounds) an appetitive behavioral habit and a drug, craving for smoking cannot be equated with craving for nicotineâ€¦. The fact that craving varies in intensity when smokers are in different situations are (sic) inconsistent with the suggestion that tobacco addiction could be reduced to craving to smoke." This is a mischaracterization of my proposal [81]. I have proposed that the diagnosis of tobacco addiction can be based on recognition of nicotine withdrawal symptoms since these are pathognomonic to tobacco addiction, i.e., no other medical or psychiatric condition causes these symptoms. In medical practice the presence of a pathognomonic symptom is by definition sufficient to establish a diagnosis. Since craving for nicotine is a characteristic symptom of nicotine withdrawal, craving attributable to withdrawal can also be used to diagnose tobacco addiction. My group has conducted over 20,000 interviews about smoking,[24,25] and there is no doubt that smokers know when they are experiencing withdrawal craving. While smoking behavior encompasses much more than a physical addiction to nicotine, identifying a physical addiction to nicotine is sufficient to identify a person with tobacco addiction. Observing that one characteristic symptom is all that is needed to recognize tobacco addiction is not equivalent to saying that "tobacco addiction could be reduced to craving."

Dar and Frenk state "As nicotine addiction is a widely accepted theory for why people smoke, responders would be likely to perceive themselves as addicted to nicotine and to attribute "symptoms" such as lack of concentration and irritability to nicotine withdrawal, especially if this particular attribution is suggested by the survey items."[1] Consistent with all the arguments in their essay, the authors present no data to support this speculation. Why would youth expect to become addicted after smoking a few cigarettes when, in the world according to Dar, prolonged daily use is a prerequisite to addiction? In the real world, adolescent smokers have very little expectation that they will become addicted, and we have shown that expectations cannot account for smokers' reports of addiction symptoms [82]. If adolescent smokers' symptoms were imaginary, measures such as the HONC would not have consistently excellent psychometric properties including predictive validity in study after study [30,59-66].

Dar and Frenk argue that smokers cannot be trusted to know what symptoms they experience during nicotine withdrawal: "none of the articles we reviewed acknowledged the difficulty inherent in taking participants' causal attributions at face value."[1] Addicted smokers experience the same withdrawal symptoms every time they go too long without smoking. They can attribute their symptoms to withdrawal because those symptoms disappear immediately every time they smoke a cigarette. To argue that smokers cannot attribute their own symptoms to withdrawal is analogous to arguing that women cannot be trusted to determine if their labor contractions are painful.

Dar and Frenk opine: "When asked what exactly it was they were addicted to, participants readily answered that it is the nicotine in cigarettes. Clearly, the responders had no way of knowing this for a fact and their ready answer only proves that they believed that smoking was driven by nicotine."[1] It wasn't the taste, or the handling of the cigarette, or the image of smoking they were addicted to, they said it was the nicotine. If alcoholics were asked "what is it about beer that you are addicted to" we would accept an answer of "the alcohol" without requiring that the subject hold a degree in psychopharmacology.

Dar and Frenk argue "Moreover, a consequence of reducing nicotine dependence to subjective craving to smoke is that the results of the "hooked on nicotine" research program cannot be compared to results of studies that use the conventional, DSM or ICD conceptualization of nicotine dependence. In other words, this conception of addiction is so removed from the rest of the field's as to render the "hooked on nicotine" research program practically incommensurable with other relevant research."[1] First, my proposal for a new approach to diagnosis is grossly oversimplified and mischaracterized by equating it with "subjective craving."[81] Second, this new approach to diagnosis was not used in any study concerning the early onset of addiction, so it is illogical to argue that it renders all previous work on early onset irrelevant to tobacco researchers. Third, Dar and Frenk seem to be arguing here that research that contradicts the conventional wisdom as embodied in the DSM or the ICD is irrelevant and should be ignored by the rest of the field. This might explain why so many scientifically indefensible hypotheses remain so popular.

Dar and Frenk start with the assumption that the hypothetical conceptualization of tobacco addiction presented by the DSM and ICD is the correct one. They then argue that data that contradict the DSM are so far removed from the DSM that they are irrelevant. Rather than rejecting a conceptualization of tobacco addiction when it is contradicted by all available data, Dar and Frenk recommend that data that contradict one's theory should be declared irrelevant.

The research investigating the onset of addiction employs traditional clinical research methods. People who have the disease are interviewed to ascertain the symptoms of the disease and the clinical course of the illness. By studying many individual cases and confirming their reports with data from large national surveys, the manner in which tobacco addiction develops has been very well established over the past 15 years. The publication of the first reports of early addiction in 2000 contradicting the threshold theory provided the field of tobacco research with an opportunity. Researchers could jump on this data as a clue toward making new discoveries, or they could ignore it. A handful of researchers have pursued this lead and as a result we now have an evidence-based description of tobacco addiction. However, many workers in this field have actively searched for excuses to dismiss or ignore the data, such as by embracing definitions of tobacco addiction that define away the possibility of early diagnosis. As a practicing physician, I can identify the typical symptoms and course of each new seasonal flu two weeks into the flu season. It is an unfortunate testimony to the state of tobacco research that the typical symptoms and course of tobacco addiction are still considered controversial 10 years after they were first described [5].

## Conclusion

For 4 decades, the threshold theory has dominated the field of tobacco research. The central tenet of this model is that prolonged moderate daily smoking is required to trigger the onset of tobacco addiction. An examination of the historical record indicates that although this theory was presented as fact, it was never supported by data. Likewise, the contention that the DSM and ICD criteria are valid measures of tobacco addiction is an unproven hypothesis. The validity of these diagnostic tests was never established prior to, or since their initial publication 3 decades ago [48,49].

Only over the past 15 years has research focused on developing an evidence-based description of the clinical course of tobacco addiction and its diagnosis by studying real smokers. A substantial body of research has been built through the use of thoroughly validated measures of tobacco addiction symptoms. The accumulated evidence comes from studies employing a variety of complimentary research methods including case histories, focus groups, experimental studies, longitudinal interview studies, and national surveys. This research has not been conducted as a coordinated "hooked on nicotine" program, but by independent researchers scattered around the world using a variety of old and new measures. The results have not been mixed. All relevant data indicate that symptoms of addiction develop during nondaily smoking. All relevant data indicate that individuals who do not maintain threshold levels of nicotine in the blood can experience all of the symptoms of nicotine withdrawal. All relevant data indicate that the onset of withdrawal symptoms can be delayed by several days or more in nondaily smokers. All relevant data indicate that the duration of relief from withdrawal that smokers obtain from smoking a cigarette shrinks as tolerance develops. Based upon this confluence of evidence, the clinical features and natural history of tobacco addiction have been established in considerable detail, and in every aspect, reality contradicts what tobacco researchers were taught by their mentors.

For 4 decades the field of tobacco research has embraced a fictitious description of tobacco addiction based on the threshold theory. For 4 decades addiction theories were built upon this fictitious vision. Now the vision and theories face the test of reality as data pour in from dozens of studies involving many tens of thousands of real smokers. The scientific method requires that hypotheses be rejected when they are contradicted by the data, but Dar and Frenk argue that the data should be declared irrelevant when they contradict popular concepts. To the degree that the DSM and the ICD cannot be reconciled with clinical data on tobacco addiction, it is the DSM and ICD that are irrelevant, not the experiences of smokers in the real world.

Our new detailed knowledge about the clinical course of tobacco addiction makes it possible to base a diagnosis on a clinician's recognition of its characteristic features [81]. But others argue that smokers do not know what tobacco addiction is, that smokers who say they are addicted but do not meet DSM criteria are mistaken: they are not addicted at all. Every year, the symptoms of each new strain of flu are determined solely by asking the people who have it. The argument by Dar and Frenk that, based on general principals of psychological research, smokers are incapable of telling us what the symptoms of tobacco addiction are, is preposterous and in conflict with how the symptoms of every disease have been established since the dawn of medicine. The need to argue that smokers cannot serve as a reliable source of data about their own disease arises because the idea that the characteristics of tobacco addiction are defined by nature and recognized by smokers is antithetical to a 30-year-old tradition that holds that tobacco addiction is whatever the DSM and ICD define it to be based on the prevailing school of thought.

In other fields of science, hypotheses are discarded as emerging data reveal that they are misdirected, but for some inexplicable reason, in the field of tobacco research, people such as Dar and Frenk dig in their heels to defend the indefensible. Against a growing mountain of empirical research, Dar and Frenk base their arguments on general principles of psychological research and their own biased concepts of tobacco addiction, but cite very little empirical smoking research to support their arguments. In an attempt to undermine the collective credibility of all researchers who have produced data that contradict the current wisdom, they portray independent researchers as being part of an organized "program." They then grossly misrepresent the ideas and claims of researchers to make them look ridiculous and extreme. They seek to call into question the validity of an entire body of research based on their own erroneous interpretation of data from a few outliers. Although they label their paper a review, they find it convenient to ignore the 99% of the published data that refute their arguments. They accuse fine Canadian researchers of committing methodological errors and demonstrating bias in their data analysis when in fact it is Dar and Frenk who reveal their ignorance of basic concepts such as susceptibility and the scoring of a standardized measure. All of this is in an attempt to protect the current wisdom from the application of the scientific method.

## Abbreviations

CPD: cigarettes per day; DSM: Diagnostic and Statistical Manual; HONC: Hooked on Nicotine Checklist; ICD: International Classification of Diseases;

## Competing interests

The author declares that they have no competing interests.

## Author's Information

JRD is a family physician and Professor of Family Medicine and Community Health at the University of Massachusetts Medical School. He has been conducting research on tobacco and health for 30 years. He is an associate editor for BMC Public Health.

## References

[B1] Dar R Frenk H Can one puff really make an adolescent addicted to nicotine? A critical review of the literature Harm Reduction 2010 in press 10.1186/1477-7517-7-28 PMC2992488 21067587

[B2] American Psychiatric Association Diagnostic and Statistical Manual of Mental Disorders: DSM-III 1980 Third Washington, DC: American Psychiatric Association

[B3] American Psychiatric Association Diagnostic and Statistical Manual of Mental Disorders, 3rd Edition, Revised 1987 Washington, DC: American Psychiatric Association

[B4] American Psychiatric Association Diagnostic and Statistical Manual of Mental Disorders: DSM-IV-TR, Fourth Edition 1994 Washington, DC: American Psychiatric Association

[B5] DiFranza JR Rigotti NA McNeill AD Ockene JK Savageau JA St Cyr D Coleman M Initial symptoms of nicotine dependence in adolescents Tob Control 2000 9 313 319 10.1136/tc.9.3.313 10982576 PMC1748379

[B6] Russell M Cigarette smoking: natural history of a dependence disorder Br J Med Psychol 1971 44 1 16 5101052 10.1111/j.2044-8341.1971.tb02141.x

[B7] Russell M Cigarette dependence: I-nature and classification Br Med J 1971 2 330 331 10.1136/bmj.2.5757.330 5575243 PMC1796044

[B8] Russell M The smoking habit and its classification The Practitioner 1974 212 791 800 4424219

[B9] Russell M Smoking problems: an overview NIDA Res Monogr 1977 17 13 33 417250

[B10] Shiffman S Tobacco "chippers": individual differences in tobacco dependence Psychopharmacology (Berl) 1989 97 539 547 10.1007/BF00439561 2498951

[B11] Shiffman S Refining models of dependence: Variations across persons and situations Br J Addict 1991 86 611 615 10.1111/j.1360-0443.1991.tb01817.x 1859928

[B12] Shiffman S Paty J Kassel J Gnys M Zettler-Segal M Smoking behavior and smoking history of tobacco chippers Experimental and Clinical Pschopharmacology 1994 2 126 142 10.1037/1064-1297.2.2.126

[B13] Shiffman S Paty JA Gnys M Kassel JD Elash C Nicotine withdrawal in chippers and regular smokers: subjective and cognitive effects Health Psychol 1995 14 301 309 10.1037/0278-6133.14.4.301 7556033

[B14] Benowitz NL Henningfield JE Establishing a nicotine threshold for addiction N Engl J Med 1994 331 123 125 10.1056/NEJM199407143310212 7818638

[B15] Benowitz N Jacob P Nicotine and cotinine elimination pharmacokinetics in smokers and nonsmokers Clin Pharmacol Ther 1993 53 316 323 10.1038/clpt.1993.27 8453850

[B16] McNeill AD West R Jarvis MJ Jackson P Bryant A Russell MAH Cigarette withdrawal symptoms in adolescent smokers Psychopharmacology (Berl) 1986 90 533 536 10.1007/BF00174074 3101108

[B17] Goddard E Why children start smoking Book Why children start smoking (Editor ed.^eds.). City 1990

[B18] Barker D Reasons for tobacco use and symptoms of nicotine withdrawal among adolescent and young adult tobacco users-United States, 1993 MMWR Morbidity and Mortality Weekly Report 1994 43 745 750 7935305

[B19] O'Loughlin J Kishchuck N DiFranza J Tremblay M Paradis G The hardest thing is the habit: a qualitative investigation of adolescent smokers' experience of nicotine dependence Nicotine & Tobacco Research 2002 4 201 209 10.1080/14622200210123000 12028853

[B20] O'Loughlin J DiFranza J Tyndale RF Meshefedjian G McMillan-Davey E Clarke PB Hanley J Paradis G Nicotine-dependence symptoms are associated with smoking frequency in adolescents Am J Prev Med 2003 25 219 225 10.1016/S0749-3797(03)00198-3 14507528

[B21] Riedel BW Robinson LA Klesges RC McLain-Allen B Ethnic differences in smoking withdrawal effects among adolescents Addict Behav 2003 28 129 140 10.1016/S0306-4603(01)00220-9 12507532

[B22] Strong DR Kahler CW Ramsey SE Abrantes A Brown RA Nicotine withdrawal among adolescents with acute psychopathology: An item response analysis Nicotine & Tobacco Research 2004 6 547 557 10.1080/14622200410001696484 15203788

[B23] An L Lein E Bliss R Pallonen U Hennrikus D Farley D Hertel A Perry C Lando H Loss of autonomy over nicotine use among college social smokers10th Annual Meeting of the Society for Research on Nicotine and Tobacco 2004 POS2 035

[B24] DiFranza JR Savageau JA Rigotti NA Fletcher K Ockene JK McNeill AD Coleman M Wood C Development of symptoms of tobacco dependence in youths: 30 month follow up data from the DANDY study Tob Control 2002 11 228 235 10.1136/tc.11.3.228 12198274 PMC1759001

[B25] DiFranza J Savageau J Fletcher K O'Loughlin J Pbert L Ockene J McNeill A Hazelton J Friedman K Dussault G Symptoms of tobacco dependence after brief intermittent use -The Development and Assessment of Nicotine Dependence in Youth-2 Arch Pediatr Adolesc Med 2007 161 704 710 10.1001/archpedi.161.7.704 17606835

[B26] Kandel D Hu M-C Grieisler P Schaffran C On the development of nicotine dependence in adolescence Drug Alcohol Depend 2007 91 26 39 10.1016/j.drugalcdep.2007.04.011 17553635 PMC2042038

[B27] Savageau J Mowery P DiFranza J Symptoms of diminished autonomy over cigarettes with non-daily use International Journal of Environmental Research and Public Health 2009 6 25 35 10.3390/ijerph6010025 19440267 PMC2672327

[B28] Dierker L Mermelstein R Early emerging nicotine-dependence symptoms: a signal of propensity for chronic smoking behavior in adolescents J Pediatr 2010 156 818 822 10.1016/j.jpeds.2009.11.044 20097354 PMC3021919

[B29] O'Loughlin J Gervais A Dugas E Meshefedjian G Milestones in the process of cessation among novice adolescent smokers Am J Public Health 2009 99 499 504 10.2105/AJPH.2007.128629 18633080 PMC2661454

[B30] Wellman R DiFranza J Wood C Tobacco chippers report diminished autonomy over tobacco use Addict Behav 2006 31 717 721 10.1016/j.addbeh.2005.05.043 15979812

[B31] Prokhorov A Hudmon K Cinciripini P Marani S "Withdrawal symptoms" in adolescents: a comparison of former smokers and never-smokers Nicotine & Tobacco Research 2005 7 909 913 10.1080/14622200500266114 16298726

[B32] Rubinstein M Benowitz N Auerback G Moscicki A Withdrawal in adolescent light smokers following 24-hour abstinence Nicotine & Tobacco Research 2009 11 185 189 10.1093/ntr/ntn028 PMC2658900 19246428

[B33] Scragg R Wellman RJ Laugesen M DiFranza JR Diminished autonomy over tobacco can appear after the first cigarette Addict Behav 2008 33 689 698 10.1016/j.addbeh.2007.12.002 18207651

[B34] Caraballo R Novak S Asman K Linking quantity/frequency profiles of cigarette smoking to the presence of nicotine dependence symptoms among adolescent smokers: findings from the 2004 National Youth Tobacco Survey Nicotine & Tobacco Research 2009 11 49 57 10.1093/ntr/ntn008 19246441

[B35] DiFranza J Ursprung W Carlson A New insights into the compulsion to use tobacco from a case series J Adolesc 2010 33 209 214 10.1016/j.adolescence.2009.03.009 19406464

[B36] Hughes JR Effects of abstinence from tobacco: valid symptoms and time course Nicotine & Tobacco Research 2007 9 315 327 10.1080/14622200701188919 17365764

[B37] DiFranza J Ursprung W The latency to the onset of nicotine withdrawal: a test of the Sensitization-Homeostasis Theory Addict Behav 2008 33 1148 1153 10.1016/j.addbeh.2008.04.011 18547736

[B38] Fernando W Wellman R DiFranza J The relationship between level of cigarette consumption and latency to the onset of retrospectively reported withdrawal symptoms Psychopharmacology (Berl) 2006 188 335 342 10.1007/s00213-006-0497-x 16953390

[B39] Ursprung S Morello P Gershenson B DiFranza J Development of a measure of the latency to needing a cigarette Journal of Adolescent Health 2010 in press 10.1016/j.jadohealth.2010.07.011 21402261

[B40] Benowitz N Nicotine Addiction N Engl J Med 2010 362 2295 2303 10.1056/NEJMra0809890 20554984 PMC2928221

[B41] Jarvik ME Madsen DC Olmstead RE Iwamoto-Schaap PN Elins JL Benowitz NL Nicotine blood levels and subjective craving for cigarettes Pharmacology Biochemistry and Behavior 2000 66 553 558 10.1016/S0091-3057(00)00261-6 10899369

[B42] Schuh KJ Stitzer ML Desire to smoke during spaced smoking intervals Psychopharmacology (Berl) 1995 120 289 295 10.1007/BF02311176 8524976

[B43] US Department of Health and Human Services Preventing Tobacco Use Among Young People, A Report of the Surgeon General 1994 Public Health Service, Centers for Disease Control and Prevention, Office on Smoking and Health

[B44] Wellman R Sugarman D DiFranza J Winickoff J The extent to which tobacco marketing and tobacco use in films contribute to children's use of tobacco: a meta-analysis Arch Pediatr Adolesc Med 2006 160 1285 1296 10.1001/archpedi.160.12.1285 17146027

[B45] Sargent JD Beach ML Adachi-Mejia AM Gibson JJ Titus-Ernstoff LT Carusi CP Swain SD Heatherton TF Dalton MA Exposure to movie smoking: its relation to smoking initiation among US adolescents Pediatrics 2005 116 1183 1191 10.1542/peds.2005-0714 16264007

[B46] Doubeni C Li W Fouayzi H DiFranza J Perceived accessibility of cigarettes among youth Am J Prev Med 2009 36 239 242 10.1016/j.amepre.2008.11.006 19162433

[B47] DiFranza JR Savageau JA Fletcher K Pbert L O'Loughlin J McNeill AD Ockene JK Friedman K Hazelton J Wood C Susceptibility to nicotine dependence: the Development and Assessment of Nicotine Dependence in Youth-2 Pediatrics 2007 120 e974 e983 10.1542/peds.2007-0027 17908753

[B48] DiFranza J Ursprung WW A systematic review of the International Classification of Diseases criteria for the diagnosis of tobacco dependence Addict Behav 2010 35 805 810 10.1016/j.addbeh.2010.04.002 20493638

[B49] DiFranza J Ursprung W Lauzon B Bancej C Wellman R Ziedonis D Kim S Gervais A Meltzer B McKay C A systematic review of the Diagnostic and Statistical Manual Diagnostic criteria for nicotine dependence Addict Behav 2010 35 373 382 10.1016/j.addbeh.2009.12.013 20056335

[B50] John U Meyer C Rumpf HJ Hapke U Depressive disorders are related to nicotine dependence in the population but do not necessarily hamper smoking cessation The Journal of clinical psychiatry 2004 65 169 10.4088/JCP.v65n0205 15003069

[B51] Pergadia ML Heath AC Martin NG Madden PA Genetic analyses of DSM-IV nicotine withdrawal in adult twins Psychol Med 2006 36 963 972 10.1017/S0033291706007495 16749946

[B52] John UM Rumpf C Schumann HJ Hapke AU Consistency or change in nicotine dependence according to the Fagerstrom Test for Nicotine Dependence over three years in a population sample J Addict Dis 2005 24 85 100 10.1300/J069v24n01_08 15774413

[B53] Cottler LB Schuckit MA Helzer JE Crowley T Woody G Nathan P Hughes J The DSM-IV field trial for substance use disorders: major results Drug Alcohol Depend 1995 38 59 69 discussion 71-83 10.1016/0376-8716(94)01091-X 7648998

[B54] Hughes JR Oliveto AH Riggs R Kenny M Liguori A Pillitteri JL MacLaughlin MA Concordance of different measures of nicotine dependence: two pilot studies Addict Behav 2004 29 1527 1539 10.1016/j.addbeh.2004.02.031 15451122

[B55] Garvey A Bliss R Hitchkock J Heinold J Rosner B Predictors of smoking relapse among self-quitters: a report from the normative aging study Addict Behav 1992 17 367 377 10.1016/0306-4603(92)90042-T 1502970

[B56] Donny EC Dierker LC The absence of DSM-IV nicotine dependence in moderate-to-heavy daily smokers Drug Alcohol Depend 2007 89 93 96 10.1016/j.drugalcdep.2006.11.019 17276627 PMC1924493

[B57] John U Meyer C Hapke U Rumpf HJ Schumann A Nicotine dependence, quit attempts, and quitting among smokers in a regional population sample from a country with a high prevalence of tobacco smoking Prev Med 2004 38 350 10.1016/j.ypmed.2003.11.003 14766119

[B58] DiFranza J Richmond J Let the children be heard: Lessons from studies of the early onset of tobacco addiction Pediatrics 2008 121 623 624 10.1542/peds.2007-3696 18310213

[B59] Wheeler KC Fletcher KE Wellman RJ DiFranza JR Screening adolescents for nicotine dependence: the Hooked On Nicotine Checklist J Adolesc Health 2004 35 225 230 15313504 10.1016/j.jadohealth.2003.10.004

[B60] Huang C Cheng C Lin H Lu C Psychometric testing of the Chinese version of the Hooked on Nicotine Checklist in adolescents J Adolesc Health 2009 45 281 285 10.1016/j.jadohealth.2009.02.012 19699424

[B61] Kleinjan M van den Eijnden RJ van Leeuwe J Otten R Brug J Engels RC Factorial and convergent validity of nicotine dependence measures in adolescents: toward a multidimensional approach Nicotine & Tobacco Research 2007 9 1109 1118 10.1080/14622200701488459 17978984

[B62] O'Loughlin J DiFranza J Tarasuk J Meshefedjian G McMillan-Davey E Paradis G Tyndale RF Clarke P Hanley J Assessment of nicotine dependence symptoms in adolescents: a comparison of five indicators Tob Control 2002 11 354 360 10.1136/tc.11.4.354 12432161 PMC1747676

[B63] Wellman R DiFranza J Pbert L Fletcher K Young M Flint A Druker S A comparison of the psychometric properties of the Hooked on Nicotine Checklist and the Modified FagerstrÃ¶m Tolerance Questionnaire Addict Behav 2006 31 486 495 10.1016/j.addbeh.2005.05.031 15993004

[B64] Wellman R DiFranza J Savageau J Godiwala S Friedman K Hazelton J Measuring adults' loss of autonomy over nicotine use: The Hooked on Nicotine Checklist Nicotine & Tobacco Research 2005 7 157 161 10.1080/14622200412331328394 15804688

[B65] Wellman R McMillen R DiFranza J Assessing College Students' Autonomy over Smoking with the Hooked on Nicotine Checklist J Am Coll Health 2008 56 549 553 10.3200/JACH.56.5.549-554 18400667

[B66] Wellman RJ Savageau JA Godiwala S Savageau N Friedman K Hazelton J DiFranza JR A comparison of the Hooked on Nicotine Checklist and the Fagerstrom Test of Nicotine Dependence in adult smokers Nicotine & Tobacco Research 2006 8 575 580 10.1080/14622200600789965 16920655

[B67] DiFranza J Hooked from the first cigarette J Fam Pract 2007 56 1017 1022 18053441

[B68] DiFranza J Hooked from the first cigarette Sci Am 2008 May 82 87 10.1038/scientificamerican0508-82 18444329

[B69] DiFranza J Riggs N Pentz M Time to re-examine old definitions of nicotine dependence Nicotine & Tobacco Research 2008 10 1109 1111 10.1080/14622200802097589 18584475

[B70] Audrain-McGovern J Rodriguez D Tercyak KP Cuevas J Rodgers K Patterson F Identifying and characterizing adolescent smoking trajectories Cancer Epidemiol Biomarkers Prev 2004 13 2023 2034 15598757

[B71] Riggs N Chou C-P Li C Pentz M Adolescent to emerging adulthood smoking trajectories: When do smoking trajectories diverge, and do they predict early adulthood nicotine dependence? Nicotine & Tobacco Research 2007 9 1147 1154 10.1080/14622200701648359 17978988

[B72] Dar R Frenk H Do smokers self-administer pure nicotine? A review of the evidence Psychopharmacology (Berl) 2004 173 18 26 10.1007/s00213-004-1781-2 15004737

[B73] Dar R Frenk H Reevaluating the nicotine delivery kinetics hypothesis Psychopharmacology (Berl) 2007 192 1 7 10.1007/s00213-007-0768-1 17404711

[B74] Dar R Kaplan R Shaham L Frenk H Euphoriant effects of nicotine in smokers: fact or artifact? Psychopharmacology (Berl) 2007 191 203 210 10.1007/s00213-006-0662-2 17235611

[B75] Dar R Serlin RC Omer H Misuse of statistical test in three decades of psychotherapy research J Consult Clin Psychol 1994 62 75 82 10.1037/0022-006X.62.1.75 8034833

[B76] Frenk H Dar R A critique of nicotine addiction 2000 New York: Kluver Academic/Plenum Publishers

[B77] Belanger M O'Loughlin J Okoli CT McGrath JJ Setia M Guyon L Gervais A Nicotine dependence symptoms among young never-smokers exposed to secondhand tobacco smoke Addict Behav 2008 33 1557 1563 10.1016/j.addbeh.2008.07.011 18760878 PMC5729007

[B78] Pierce J Choi W Gilpin E Farkas A Merritt R Validation of susceptibility as a predictor of which adolescents take up smoking in the United States Health Psychol 1996 15 355 361 10.1037/0278-6133.15.5.355 8891714

[B79] Wellman RJ DiFranza JR Savageau JA Dussault GF Short term patterns of early smoking acquisition Tob Control 2004 13 251 257 10.1136/tc.2003.005595 15333880 PMC1747885

[B80] Doubeni C Reed G DiFranza J The early course of symptom development in adolescent smokers Pediatrics 2010 125 1127 1135 10.1542/peds.2009-0238 20439592 PMC3079339

[B81] DiFranza J A new approach to the diagnosis of tobacco addiction Addiction 2010 105 381 382 10.1111/j.1360-0443.2010.03183.x 20402977

[B82] Ursprung W DiFranza S Costa A DiFranza J Might expectations explain early self-reported symptoms of nicotine dependence? Addict Behav 2008 34 227 231 10.1016/j.addbeh.2008.10.013 18986771

